# Mortality and cancer incidence in carriers of constitutional t(11;22)(q23;q11) translocations: A prospective study

**DOI:** 10.1002/ijc.32031

**Published:** 2019-01-11

**Authors:** Minouk J Schoemaker, Michael E Jones, Craig D Higgins, Alan F Wright, the UK Clinical Cytogenetics Group, Anthony J Swerdlow

**Affiliations:** ^1^ Division of Genetics and Epidemiology The Institute of Cancer Research London United Kingdom; ^2^ Faculty of Epidemiology and Population Health The London School of Hygiene and Tropical Medicine London United Kingdom; ^3^ MRC Human Genetics Unit MRC Institute of Genetics and Molecular Medicine Edinburgh United Kingdom; ^4^ Division of Breast Cancer Research The Institute of Cancer Research London United Kingdom

**Keywords:** chromosome aberrations, cytogenetics, cohort studies, epidemiology, mortality, neoplasms, breast cancer

## Abstract

The constitutional t(11;22)(q23;q11) translocation is the only recurrent non‐Robertsonian translocation known in humans. Carriers are phenotypically normal and are usually referred for cytogenetic testing because of multiple miscarriages, infertility, or having aneuploidy in offspring. A breast cancer predisposition has been suggested, but previous studies have been small and had methodological shortcomings. We therefore conducted a long‐term prospective study of cancer and mortality risk in carriers. We followed 65 male and 101 female carriers of t(11;22)(q23;q11) diagnosed in cytogenetic laboratories in Britain during 1976–2005 for cancer and deaths for an average of 21.4 years per subject. Standardised mortality (SMR) and incidence (SIR) ratios were calculated comparing the numbers of observed events with those expected from national age‐, sex‐, country‐ and calendar‐period‐specific population rates. Cancer incidence was borderline significantly raised for cancer overall (SIR = 1.56, 95% CI: 0.98–2.36, n = 22), and significantly raised for invasive breast cancer (SIR = 2.74, 95% CI: 1.18–5.40, n = 8) and *in situ* breast cancer (SIR = 13.0, 95% CI: 3.55–33.4, n = 4). Breast cancer risks were particularly increased at ages <50 (SIR = 4.37, 95% CI: 1.42–10.2 for invasive, SIR = 22.8, 95% CI: 2.76–82.5 for *in situ*). Mortality was borderline significantly raised for breast cancer (SMR = 4.82, 95% CI: 0.99–14.1) but not significantly raised for other cancers or causes. Individuals diagnosed with t(11;22)(q23;q11) appear to be at several‐fold increased breast cancer risk, with the greatest risks at premenopausal ages. Further research is required to understand the genetic mechanism involving 11q23 and 22q11 and there may be a need for enhanced breast cancer surveillance among female carriers.

## Abstract 2

**What's new?**

The constitutional translocation between chromosome bands 11q23 and 22q11 is recurrent in human populations, with highly consistent breakpoints. A breast cancer predisposition among carriers has been suggested, but previous studies have been small and had methodological shortcomings. In this first long‐term follow‐up study of site‐specific cancer and mortality risks among carriers, an increased risk of breast cancer was observed compared to the general population, with greatest risks in younger women. The results suggest that carriers of t(11;22)(q23;q11) may require enhanced surveillance for breast cancer and point to the importance of the chromosomal regions 11q23 and 22q11 in breast cancer development.

**Abbreviations**
AER: absolute excess rate
CI: confidence interval
ICD: International Statistical Classification of Diseases
SIR: Standardised incidence ratio
SMR: Standardised mortality ratio

## Introduction

The constitutional t(11;22)(q23;q11) translocation is the only recurrent non‐Robertsonian translocation known in humans, with highly consistent breakpoints.^1^, ^2^ Carriers are phenotypically normal and are usually diagnosed as a result of investigation for multiple miscarriages, infertility, or after having offspring with Emanuel syndrome (supernumeracy derivative 22 syndrome).

Investigation of mortality and cancer risks in t(11;22) carriers is important for counselling of carriers and can additionally provide insights into our understanding of cancer aetiology. A predisposition to breast cancer among female carriers has been suggested based on the presence of subjects with a history of breast cancer among relatively small clinical series of carriers,^3^, ^4^, ^5^, ^6^ although a recent pedigree study did not observe an excess of cases.^7^ Such studies have several potential methodological shortcomings including biased selection of carriers, dependence on recall of historical breast cancer diagnoses in relatives by a proband, and lack of detail on relatives to compute the expected numbers of cases.

A prospective investigation, in which subjects diagnosed with the translocation are followed up over time for subsequent cancer risk would avoid these potential biases. However, there are no such prospective studies except for one very small investigation including 16 carriers.^8^ We therefore conducted a long‐term prospective follow‐up study of carriers diagnosed across Britain and analysed site‐specific cancer incidence and cause‐specific mortality.

## Methods

We collected information on carriers diagnosed postnatally with t(11;22)(q23;q11) from all but one cytogenetic centres in Britain between 1976 and 2005 (exact years varied by centre). Carriers whose records indicated they had been ascertained because of cancer were excluded. Information on subjects was matched to the National Health Service Central Registers (NHSCRs) for England and Wales and Scotland, which provided information on deaths, emigrations and other exits from the NHS, and are effectively population registers for these countries. Permission for our study was obtained from appropriate ethics committees in the UK and the national personal information advisory group.

Follow‐up for cancer and mortality risk started at cytogenetic diagnosis and ended on the latest date event notifications were thought to be complete (31 December 2015 for incidence, 31 December 2016 for mortality), the patient's 85th birthday, date of death or date of other loss to follow‐up, whichever was earliest. The observed numbers of deaths and cancers since cytogenetic diagnosis were compared against the expected number of cases based on age‐, sex‐, country‐ and calendar‐year specific mortality and incidence rates from the general population. We computed standardised mortality (SMR) and incidence (SIR) ratios and their 95% confidence intervals (CIs) using exact methods.^9^ Where zero cases were observed, the upper bound of the confidence interval reflected the 97.5% distribution to make the boundary comparable to those of estimates with non‐zero observations. We calculated absolute excess rates (AERs) by subtracting the expected from the observed numbers of deaths, dividing by person‐years at risk and multiplying by 100,000. We analysed neoplasms classified as malignant according to the ICD 9th revision^10^ except that we additionally analysed *in situ* breast cancer and that analyses of nervous system tumours included non‐malignant registrations. Analyses were conducted for all subjects combined and, for breast cancer outcomes, by attained age <50, ≥50 years as a proxy for menopausal status in females. In order to investigate the possibility that mortality or cancer incidence might have been biased because some subjects were cytogenetically tested as a consequence of a prior illness, analyses were repeated after excluding from follow‐up the first 36 months after cytogenetic diagnosis.

## Results

We ascertained 217 subjects diagnosed with t(11;22)(q23;q11) at the study centres, 170 of whom could be matched on the NHSCRs because sufficient details were available. After excluding 4 subjects referred for testing because of cancer, the cohort study consisted of 166 individuals (65 males and 101 females) (Table 1). They were diagnosed at a median age of 30.9 years (range 0–84.8 years). Median follow‐up was 21.4 years per subject for mortality and 20.4 years for incidence.

**Table 1:** Characteristics of 166 subjects cytogenetically diagnosed with t(11;22)(q23;q11) during 1976–2005 (varying by cytogenetic centre) in Great Britain

Characteristic	No.	%
*Sex*		
Male	65	39.2
Female	101	60.8
*Age at cytogenetic diagnosis (years)*		
0–4	6	3.6
5–14	13	7.8
15–24	22	13.3
25–34	65	39.3
35–44	22	13.3
45–64	30	18.1
65–84	8	4.8
*Calendar year of cytogenetic diagnosis*		
1976–1979	2	1.2
1980–1989	32	19.3
1990–1999	125	75.3
2000–2005	7	4.2
*Year of birth*		
<1950	43	25.9
1950–1969	81	48.8
1970–1989	36	21.7
1990–2005	6	3.6
Total	166	100.0

During follow‐up, 20 subjects died, 134 were followed‐up to the end of the study or age 85, and 12 emigrated or exited in other ways. There were 22 incident malignancies recorded during follow‐up, a borderline significantly greater number than expected from national rates (SIR = 1.56, 95% CI: 0.98–2.36) (Table 2). There were 8 invasive and 4 *in situ* diagnoses of breast cancer in 10 subjects aged 38–82 years. Diagnoses in the two subjects with multiple registrations were at least 4 years apart. Incidence was significantly raised for invasive (SIR = 2.74, 95% CI: 1.18–5.40, AER = 155, i.e. 155 additional cases per 100,000 population *per annum*) and *in situ* (SIR = 13.0, 95% CI: 3.55–33.4, AER = 112.9) breast cancer, with all cases occurring in females. Risk of breast cancer was more greatly raised at younger ages. For invasive breast cancer, the SIR was 4.37 (95% CI: 1.42–10.2) at ages <50 and 1.69 (95% CI: 0.35–4.93) at ages ≥50 years (Table 3). Two of the invasive cases were diagnosed under age 40 years (SIR = 7.58, 95% CI: 0.92–27.4) (not in table). For *in situ* breast cancer at ages <50 years, the SIR was 22.8 (95% CI: 2.76–82.5) and risk was also significantly raised at older ages (SIR = 9.12, 95% CI: 1.10–33.0).

**Table 2:** Cancer incidence and mortality in carriers of t(11;22)(q23;q11) translocations

			Incidence			Mortality	
ICD‐9 code	Cancer site	No.	SIR (95% CI)	AER	No.	SMR (95% CI)	AER
140–171, 173–208	All malignant neoplasms^a^	22	1.56 (0.98–2.36)	241.0	9	1.44 (0.66–2.74)	81.3
150	Oesophagus	1	3.18 (0.08–17.7)	20.9	0	0 (0–12.8)	−8.5
153–154	Colon and rectum	1	0.64 (0.02–3.54)	−17.5	0	0 (0–6.11)	−17.7
162	Lung	4	2.35 (0.64–6.01)	70.1	3	2.08 (0.43–6.09)	45.9
163	Pleura	1	11.1 (0.28–62.0)	27.8	0	0 (0–129.4)	−0.84
174–175, 233.0	Breast, invasive or *in situ*	**12**	**3.72 (1.92–6.49)** **	**268.2**	3	4.82 (0.99–14.1)	70.0
174, 175	Breast, invasive	**8**	**2.74 (1.18–5.40)** *	**155.0**	3	4.82 (0.99–14.1)	69.9
233.0	Breast, *in situ*	**4**	**13.0 (3.55–33.4)** **	**112.9**	0	0 (0–∞)	0
179,182	Corpus uteri	2	5.55 (0.67–20.1)	81.4	0	0 (0–54.6)	−3.2
185	Prostate	1	0.69 (0.02–3.87)	−35.0	0	0 (0–13.4)	−21.0
189	Kidney and ureter	1	2.70 (0.07–15.1)	19.2	0	0 (0–24.9)	−4.4
200, 202	Non‐Hodgkin lymphoma	1	1.93 (0.05–10.8)	14.7	1	5.41 (0.14–30.1)	24.0
204–8	Leukaemia	1	3.15 (0.08–17.6)	20.8	0	0 (0–22.3)	−4.9
191, 192, 225, 237.5, 237.6, 237.9, 239.6	Nervous system, including benign	0	0 (0–8.91)	−12.6	1	4.34 (0.11–24.2)	22.6
196.0–199.1	Unknown primary site	0	0 (0–8.62)	−13.1	1	1.96 (0.05–10.9)	14.4

Abbreviations: AER, absolute excess rate; CI, confidence interval; ICD, International Statistical Classification of Diseases, 9th revision;^10^ SIR, standardised incidence ratio; SMR, standardised mortality ratio.

* *p* < 0.05.

** *p* < 0.001.

a The category ‘All malignant neoplasms’ included neoplasms classified as malignant according to the ICD revision 9,^10^ excluding non‐melanoma skin cancer because it is under‐ ascertained by the cancer registries.^23^ Analyses by cancer site included registrations coded to malignant, with the exception that *in situ* breast cancers and non‐malignant nervous system tumours were included where stated. 1 malignancy of ill‐defined site is not listed individually in the table.

**Table 3:** Breast cancer incidence in carriers of t(11;22)(q23;q11) translocations, by attained age

			Attained age, years				
			<50			≥50	
ICD‐9 code	Cancer site	No.	SIR (95% CI)	AER	No.	SIR (95% CI)	AER
174, 175, 233.0	Breast, invasive or *in situ*	**7**	**5.69 (2.29–11.7)** ***	**255.5**	5	2.50 (0.81–5.84)	296.4
174, 175	Breast, invasive	**5**	**4.37 (1.42–10.2)** *	**170.5**	3	1.69 (0.35–4.93)	120.6
2,330	Breast, *in situ*	**2**	**22.8 (2.76–82.5)** **	**84.7**	**2**	**9.12 (1.10–33.0)** *	**175.8**

Abbreviations: AER, absolute excess rate; CI, confidence interval; ICD, International Statistical Classification of Diseases, 9th revision;^10^ SIR, standardised incidence ratio.

* *p* < 0.05.

** *p* < 0.01.

*** *p* < 0.001.

All of the incident cancers were diagnosed at least 3 years after cytogenetic diagnosis and excluding the first 3 years of follow‐up after cytogenetic diagnosis led to slightly stronger SIRs (SIR = 1.70, 95% CI: 1.07–2.57 for all malignancies and SIR: 2.95, 95% CI: 1.27–5.81 for invasive breast cancer, not in table). SIRs for other sites were not significantly raised or reduced, but the numbers of cases were small (Table 2). There were 4 cases of lung cancer, 2 cases of corpus uteri cancer and single cases at other sites. The 2 malignancies of the corpus uteri were both endometrioid.

We did not collect information on pedigrees but based on cytogenetic centre and patient details we ascertained that our study population included at least 64 individuals with one or more relatives in the study, from 22 families. All malignancies, including the 10 breast cancer cases, appeared to have arisen in subjects from independent families.

Cancer mortality overall was not significantly raised in comparison with the general population expectation (SMR = 1.44, 95% CI: 0.66–2.74, *n* = 9 deaths), and was borderline significantly raised for invasive breast cancer (SMR = 4.82, 95% CI: 0.99–14.1, *n* = 3 deaths), but not for other cancer‐sites. The SMR for all malignancies except breast cancer was 1.07 (95% CI: 0.39–2.33). Two of the deaths from breast cancer were among women aged <50 (SMR = 11.0, 95% CI: 1.34–39.9, not in table). After excluding the first 3 years after cytogenetic diagnosis from the analysis, the SMR for breast cancer at all ages was 5.34 (95% CI: 1.10–15.6). Mortality from other causes than cancer was not raised (Table 4).

**Table 4:** Cause‐specific mortality, by ICD‐9 chapter,^10^ in carriers of t(11;22)(q23;q11) translocations

ICD‐9 Code	Cause	No. of deaths	SMR (95% CI)	AER
140–208	All malignant neoplasms	9	1.44 (0.66–2.74)	81.3
240–279	Endocrine, nutritional, metabolic, immunity	1	3.61 (0.09–20.1)	21.3
290–319	Mental disorders	0	0.0 (0–9.36)	−11.6
320–389	Diseases of the nervous system	0	0.00 (0–6.70)	−16.2
390–459	Diseases of the circulatory system	3	0.56 (0.12–1.63)	−69.7
460–519	Diseases of the respiratory system	5	2.72 (0.88–6.35)	93.0
520–579	Diseases of the digestive system	2	2.02 (0.24–7.29)	29.6
580–629	Diseases of the genitourinary system	0	0.00 (0–18.4)	−5.9
710–739	Musculoskeletal system and connective tissue	0	0.00 (0–33.1)	−3.3
740–759	Congenital anomalies	0	0.00 (0–59.7)	−1.8
800–999	Accidents and violence	0	0.00 (0–3.94)	−27.5
001–999	All causes	20	1.14 (0.70–1.77)	74.3

Abbreviations: AER, absolute excess rate; CI, confidence interval; ICD, International Statistical Classification of Diseases, 9th revision 10; SMR, standardised mortality ratio.

## Discussion

We observed that carriers were at several‐fold greater risk of breast cancer than the general population, with the greatest risk increases among young women, but there was no substantial evidence of a raised risk for other cancers. The associations were stronger when we only considered follow‐up more than 3 years after cytogenetic diagnosis, therefore making it highly unlikely that the findings are due to referral because of a pending cancer diagnosis. The only previous prospective investigation of cancer risk in carriers observed that, among 16 carriers of t(11;22)(q23:q11), one of the two subjects who developed cancer during follow‐up had breast cancer, but this did not reach statistical significance (SIR = 2.63, 95% CI: 0.30–9.50).^8^

It is unclear why the relative risk increases were greater for *in situ* than invasive breast cancer. A greater proportion of *in situ* than invasive tumours are first detected by mammographic screening, and *in situ* cancers represent a greater proportion of screen‐detected cancers among women screened at ages <50 years than older ages.^11^ If the raised risk of breast cancer were causal, we might expect carriers to be more likely to have a family history of breast cancer than the general population. It is possible therefore that risks of *in situ* breast cancer are somewhat inflated because carriers are invited for enhanced breast cancer screening due to their family history.

Previous reports of a breast cancer predisposition in t(11;22) carriers have been based on identification of individuals with a history of breast cancer in pedigree studies or case series of carriers.^3^, ^4^, ^5^, ^6^, ^7^ Some of the studies have had contradictory results, however. Lindblom *et al* observed four cases of breast cancer among 16 female carriers identified from eight pedigree families, which they estimated constituted a 10‐fold increased risk.^3^ However, the largest pedigree study to date, by Carter *et al*, did not observe an excess of breast cancer cases among 103 female carriers from 80 pedigrees ascertained for reproductive‐related reasons.^7^ Information on carrier status and breast cancer in relatives was initially obtained from a proband carrier in each family and such dependence on recall by the proband is a possible cause of bias. Some past studies are also prone to ascertainment bias if a subject or family is recruited into the investigation due to their cancer history or is referred for cytogenetic testing because of a cancer diagnosis. Furthermore, in past studies it might have been difficult to establish whether there is an excess of cases given that the expected number in the reference population depends on the number of relatives, their ages and calendar periods of follow‐up. Our study design overcomes such weaknesses due to its longitudinal design, computation of expected rates, national follow‐up and use of routinely collected cancer registrations.

The observed raised risks of breast cancer, in particular among younger women, point to importance of the 11q23 and 22q11 regions in breast cancer development. Two common regions of loss of heterozygosity have been identified in the region 11q23 in breast cancer.^12^, ^13^, ^14^ The region 22q11.2 is known to be susceptible to rearrangements^15^ and both breakpoints in t(11;22) carriers have been mapped outside gene coding regions to similar palindromic AT‐rich repeats (PATRRs).^1^, ^2^, ^6^ Intrastrand base‐pairing of AT‐rich palindromes can lead to formation of hairpin and cruciform structures that promote double strand breaks and illegitimate recombination between homologous regions on distinct chromosomes such as the 11q23 and 22q11 sites. The increased risk of breast cancer may result from a consequent ‘position effect’ of the translocation on the expression of a neighbouring breast cancer susceptibility gene(s).^16^ The 22q11.2 cannot be unambiguously assigned within a large 424 kb region; but the 11q23.3 breakpoint has been mapped to a region approximately 9 kb from APOA4,^2^ within a cluster of apolipoprotein genes. While there is no known association between APOA4 and breast cancer risk, two plausible candidates (ZNF259 and BUD13) map approximately 42 kb and 57 kb centromeric to the 11q23.3 breakpoint. Alternatively, loss of the small der (22) chromosome by nondisjunction during meiosis could also result in loss of a tumour suppressor gene copy (similar to the 11q23 loss‐of‐heterozygosity observed in breast cancer) leading to a breast cancer disposition.^6^

The number of calendar years for which we extracted records varied by centre depending on the years the centre was operational, availability of historical records and the calendar period of extraction. The availability of records for operational reasons is unlikely to have caused bias in the results. However, most carriers are not detected unless they get referred for cytogenetic testing for experiencing reproductive problems or abnormalities in offspring. Our study is therefore of patients with t(11;22) who are eventually diagnosed, but from a clinical and counselling perspective this is the group of carriers of relevance. It is possible that the raised risks of breast cancer are related to the selective forces that lead to cytogenetic diagnosis of carriers: for instance if women who get referred for cytogenetic testing are of higher socioeconomic status than those who are not referred (breast cancer rates being greater in higher socioeconomic strata).^17^ Additionally, carriers might have fertility problems, and nulliparity and delayed childbirth are risk factors for breast cancer.^18^ The latter is somewhat supported by the finding that risk of endometrial cancer, which is associated with nulliparity and infertility,^19^ was non‐significantly raised in this cohort, based on two cases. These factors are not expected to increase breast cancer risk more than twofold, however, and therefore do not explain the raised risks in full. We did not have information on whether individuals had also been tested for BRCA1 or BRCA2 mutations, but the prevalence of these mutations is low, ~2% in unselected patients diagnosed age 35 and over,^20^ and we are not aware of such mutations being more common in t(11;22) carriers. It therefore seems unlikely that the presence of BRCA1/BRCA2 mutations is the reason for the raised breast cancer risks observed in this cohort.

The translocation t(11;22)(q23;q11) represents 1.2% of all diagnosed reciprocal translocations,^8^ the latter having a frequency of 0.5–2.7 per 1,000 in the population.^21^, ^22^ Given the frequency of the translocation in the population, our prospective study comprised a relatively large population of carriers. However, a limitation of the study is that the number of carriers was still too small to investigate less common types of cancer or associations with modest risk increases. Risks of melanoma and oesophageal cancer were not increased among carriers, in contrast to the report by Carter *et al* 7; we observed one case of oesophageal cancer, but the SIR was not significantly raised. Additionally, we did not collect information on subjects’ lifestyle factors and it is possible that the carriers are not representative of the general population with respect to other factors.

In conclusion, individuals diagnosed with t(11;22)(q23;q11) showed increased rates of breast cancer compared with the general population, in particular in women aged under 50 years. The mechanistic involvement of the breakpoints on 11q23 and 22q11 in breast cancer development needs to be investigated and increased breast cancer surveillance in female carriers may be warranted.

## Authors’ contributions

AJS was the principal investigator of the study. AJS and MJS organised the collection of the national data and AFW contributed to this collection. MJS, CDH and MEJ analysed the data. MJS drafted the manuscript and all authors contributed to the interpretation of the findings. All authors contributed to the final draft.

## Members of the UK clinical Cytogenetics group

Authors and affiliations from the UK Clinical Cytogenetics Group are as follows: Paul J Batstone (Inverness Genetics Laboratory, Raigmore Hospital, Inverness, UK); Nick Bown (Northern Genetics Service laboratories, Newcastle upon Tyne, UK); Catherine Delmege (Bristol Genetics Laboratory, North Bristol NHS Trust, Southmead Hospital, Bristol, UK); Carolyn Campbell (Oxford Genetics Laboratory, Oxford University Hospitals NHS Foundation Trust, Oxford, UK); Angela Douglas (Regional Genetics Laboratory, Liverpool Women's NHS Foundation Trust, Liverpool, UK); David Duckett (Leicestershire Cytogenetics Centre, Leicester Royal Infirmary, Leicester, UK); Sandra Edwards (Cytogenetics Laboratory, Norfolk & Norwich University Hospital Foundation Trust, Norwich, UK); Lorraine Gaunt (Genomic Diagnostics Laboratory, Manchester Centre for Genomic Medicine, Manchester University NHS Foundation Trust, Manchester, UK); Michael Griffiths (West Midlands Regional Genetics Laboratory, Birmingham Women's and Children's NHS Foundation Trust, Birmingham, UK); Richard Hall (South East Thames Regional Genetics Laboratory, Guy's Hospital, London, UK); Christine Maliszewska (East Scotland Human Genetic Laboratories, Ninewells Hospital and Medical School, Dundee, UK); Katherine Martin (Nottingham Regional Genetics Laboratories, Nottingham University Hospitals NHS Trust, Nottingham, UK); Karen Marks (South West Thames Regional Genetics Service, St George's Hospital, London, UK); Lucy Platts (North East Thames Regional Genetics Laboratory, Great Ormond Street Hospital NHS Foundation Trust, London, UK); Andrew Pearce (South East Scotland Regional Genetics Centre, Western General Hospital, Edinburgh, UK); Paul Roberts (Yorkshire Regional Genetics Service, St James's University Hospital, Leeds, UK); Kath Smith (Sheffield Diagnostic Genetics Service, Sheffield Children's NHS Foundation Trust, Sheffield, UK); David Stevenson (North of Scotland Medical Genetic Service, Aberdeen Royal Infirmary, Aberdeen, UK); Peter Thompson (Institute of Medical Genetics, University Hospital of Wales, Cardiff, UK); Christine Waterman (Wessex Regional Genetics Laboratory, Salisbury NHS Foundation Trust, Salisbury, UK); Catherine Waters (North West Thames Regional Genetics Service, Northwick Park & St Marks NHS Trust, Middlesex, UK). We additionally thank the following centres for providing data: West of Scotland Regional Genetics Service, Yorkhill NHS Trust, Glasgow, UK; East Anglia Regional Genetics Service, Addenbrookes Hospital, Cambridge, UK.
